# Probiotic co-supplementation with absorbent smectite for pancreatic beta-cell function in type 2 diabetes: a secondary-data analysis of a randomized double-blind controlled trials

**DOI:** 10.3389/fendo.2024.1276642

**Published:** 2024-02-08

**Authors:** Maryana Savytska, Dmytro Kyriienko, Ganna Zaychenko, Danylo Ostapchenko, Tetyana Falalyeyeva, Nazarii Kobyliak

**Affiliations:** ^1^ Normal Physiology Department, Danylo Halytsky Lviv National Medical University, Lviv, Ukraine; ^2^ Kyiv City Clinical Endocrinology Center, Kyiv, Ukraine; ^3^ Pharmacology Department, Bogomolets National Medical University, Kyiv, Ukraine; ^4^ Educational-Scientific Center “Institute of Biology and Medicine” Taras Shevchenko National University of Kyiv, Kyiv, Ukraine; ^5^ Medical Laboratory CSD, Kyiv, Ukraine; ^6^ Endocrinology Department, Bogomolets National Medical University, Kyiv, Ukraine

**Keywords:** probiotics, smectite, absorbent, gut microbiota, type 2 diabetes, pancreatic β-cells, β-cells dysfunction

## Abstract

**Introduction:**

There is growing evidence from animal and clinical studies suggesting probiotics can positively affect type 2 diabetes (T2D). In a previous randomized clinical study, we found that administering a live multistrain probiotic and absorbent smectite once a day for eight weeks to patients with T2D could reduce chronic systemic inflammatory state, insulin resistance, waist circumference and improve the glycemic profile. However, there is a lack of evidence supporting the efficacy of probiotic co-supplementation with absorbent smectite on pancreatic β-cell function in T2D.

**Aim:**

This secondary analysis aimed to assess the effectiveness of an alive multistrain probiotic co-supplementation with absorbent smectite *vs* placebo on β-cell function in T2D patients.

**Material and methods:**

We performed a secondary analysis on a previously published randomized controlled trial (NCT04293731, NCT03614039) involving 46 patients with T2D. The main inclusion criteria were the presence of β-cell dysfunction (%B<60%) and insulin therapy alone or combined with oral anti-diabetic drugs. The primary outcome was assessing β-cell function as change C-peptide and %B.

**Results:**

We observed only a tendency for improving β-cell function (44.22 ± 12.80 *vs* 55.69 ± 25.75; р=0.094). The effectiveness of the therapy probiotic-smectite group was confirmed by fasting glycemia decreased by 14% (p=0.019), HbA1c – 5% (p=0.007), HOMA-2 – 17% (p=0.003) and increase of insulin sensitivity by 23% (p=0.005). Analysis of the cytokine profile showed that statistical differences after treatment were in the concentration of both pro-inflammatory cytokines: IL-1β (22.83 ± 9.04 *vs* 19.03 ± 5.57; p=0.045) and TNF-α (31.25 ± 11.32 *vs* 26.23 ± 10.13; p=0.041).

**Conclusion:**

Adding a live multistrain probiotic and absorbent smectite supplement slightly improved β-cell function and reduced glycemic-related parameters in patients with T2D. This suggests that adjusting the gut microbiota could be a promising treatment for diabetes and warrants further investigation through more extensive studies.

## Introduction

Diabetes is a chronic disease that arises when the β-cells in the pancreas fail to produce sufficient insulin or when the body cannot effectively use the insulin it generates. Around 95% of people with diabetes worldwide have type 2 (T2D). Between 2000 and 2019, there was a 3% increase in age-standardized mortality rates from diabetes (1). T2D belongs to a group of metabolic diseases inherent in hyperglycemia on the background of insulin resistance (IR) and reduction of insulin secretion (2, 3). Chronic hyperglycemia can damage and dysfunctional various organs, such as retinopathy, nephropathy, metabolic and cardiovascular diseases (2, 4). Even though many standard and non-standard schemes for treating T2D have been developed today, the number of new cases does not decrease but instead increases (5). Often, a prerequisite for T2D is obesity and disrupting the microbiota in the large intestine (6, 7).

The gut microbiota is gaining meaningful scientific interest concerning obesity and different associated metabolic disorders to understand obesity’s etiology better and find modern methods for its prevention and/or treatment (8, 9). It is established that obesity and T2D are characterized by a chronic state of low-grade inflammation with IR (10). Inflammation is a nonspecific biological response of the immune system to pathogens, toxins, and damaged cells (11). This acute or chronic reaction can lead to tissue damage (12). For several decades, it has been believed that increased inflammatory tone significantly affects glucose metabolism. Therefore, sorbents are indispensable in the fight against chronic inflammation in patients with T2D (13). Chronic inflammation is accompanied by oxidative stress, which results in β-cell dysfunction and α-cell expansion in the pancreas. This leads to the progression of T2D in obese subjects and gut microbiota influences the secretion of these molecules (14–16). Depending on the strain, bacteria can indirectly stimulate pro-inflammatory cytokine production by the host through their metabolites or reduce inflammation by synthesizing anti-inflammatory substances (17). So today, much animal and clinical data suggest the beneficial effects of probiotics in T2D, which mainly focus on their impact on IR, anthropometric parameters, glycemic control and markers of chronic systemic inflammation (18).

Our previous randomized clinical study established that a live multistrain probiotic and absorbent smectite once a day for eight weeks to patients with T2D could reduce chronic systemic inflammatory state, IR, waist circumference and improve glycemic profile (19). Also, it was established that smectite, due to its ability to bind endo- and exotoxins and its capacity to restore the barrier properties of human intestinal cell monolayers, may be beneficial when supplemented with probiotics for NAFLD/NASH development (13, 20).

This work aimed to assess the effectiveness of an alive multistrain probiotic co-supplementation with absorbent smectite *vs* placebo on β-cell function in T2D patients.

## Materials and methods

### Ethics statement

We conducted a secondary analysis of previously published randomized clinical trials (RCTs) (NCT04293731, NCT03614039) (19, 20). Patient selection was conducted at the Kyiv City Clinical Endocrinology Centre, Ukraine. The primary research protocols were approved by the local Ethics Committee (protocol 2/5_2015 and 2017.19/4) and put into practice on the basis of the Declaration of Helsinki (1975). Before beginning the RCT, the study’s objectives and procedures were comprehensively explained to the participants. After the discussion, all patients voluntarily gave their informed consent by signing the required paperwork.

### Inclusion criteria

The inclusion criteria were the following: adult participants (aged 18 to 75) with proven T2D diagnosis based on the criteria of the American Diabetes Association (plasma glucose in fasting state ≥7.0 mmol/l; plasma glucose at random measuring ≥11.1 mmol/l; HbA1c ≥6.5% or glucose > 11.1 mmol/l 2 hours after tolerance test with 75 g of glucose) (21); presence of pancreatic β-cell dysfunction which defined as %B<60% and treatment with insulin therapy alone or in combination with oral anti-diabetic drugs (metformin and/or sulphonylureas) in a stable dose for at least 3 months prior to randomization; HbA1c level 6.5 to 10.0%; a signed informed consent.

### Exclusion criteria

The main exclusion criteria were the presence of T1D; intake of anti-diabetic drugs except for those specified in the inclusion criteria (pioglitazone, glucagon-like peptide (GLP-1) analogs, dipeptide-peptidase 4 (DPP-4) inhibitors, etc.); severe diabetes-related complications at screening (ie, end-stage diabetic kidney disease, neuropathy requiring pharmacological treatment, proliferative retinopathy, autonomic neuropathy); regular intake of probiotics, prebiotics or antibiotics for 3 months prior the inclusion; previously diagnosed allergy to probiotics; gastrointestinal disorders including food allergy, gluten-sensitive enteropathy, ulcerative colitis; an uncontrolled cardiovascular or respiratory disease, an active malignant tumor or chronic infections; participation in another clinical trial; pregnancy or lactation.

### Study design

The background of secondary analysis was previous RCT were the administration of Probiotic-Smectite was not accompanied by significant changes in the functional activity of β-cells (% B) according to the HOMA2 model (19). The lack of a valid difference and multidirectionality of changes, in the authors’ opinion, was preconditioned by the trial design. This is because when the study was scheduled, the functional activity of β-cells was not considered to be an individual preset criterion of inclusion/exclusion and was only assessed as additional parameter in terms of secondary endpoints. For this reason, patients with both hyper and dysfunction of β-cells of various intensities were included in the trial, thus influencing the final interpretation of the results according to this parameter (22). The current study aimed to assess the effectiveness of Probiotic-Smectite combination *vs* placebo in T2D patients with primary β-cell dysfunction. For this purpose, we conducted secondary analysis of published RCTs (19, 20) and after repeated database analysis included patients who responded to updated inclusion/exclusion criteria.

The RCT included T2D patients, who were randomly with an allocation ratio 1:1 prescribed either probiotic or a placebo for 8 weeks. Randomization was double-blind and carried out by a statistical expert with blocks of four using a computer-generated list at www.randomization.com. The groups were homogeneous in terms of age, gender, and diagnosis. The co-investigators distributed the sachets among the participants according to their groups. The group allocation was blind both for the participants and the researchers. In addition to that, with view of supporting the double-blind design the statistics expert did not know the distribution of the participants between the study groups. The code was broken after the analysis had been completed and the database had been closed.

The preliminary randomization period was developed to reduce the effects of diet changes upon metabolic markers. For this purpose, two weeks before randomization all patients were offered to have a one-time session with a dietician to modify their lifestyle. The nutrition program included a corrective diet and drinking regime (natural water daily norm 30-40 ml/kg). Patients were provided with a list of recommended and prohibited products. Any cooking method was recommended except the fry. The last meal was 1.5-2 hours before bedtime. In addition to that the participants were offered to continue their usual intake of anti-diabetic drugs and get a standard average physical exercise for at least 1 hour a day.

Throughout the study, follow-up phone visits were conducted every two weeks to ensure that participants were complying with the protocol requirements and to monitor for any adverse events. In the study, participants who experienced minor negative reactions were given the option to continue or stop taking the supplements, but they still had to attend follow-up appointments. However, patients who reported serious adverse events, such as diarrhea, nausea/vomiting, or sepsis, were not included in the final analysis if they had changes in their previous therapy or had taken antibiotics.

To assess patient compliance, the remaining supplement sachets were counted and an investigator directly asked participants about their adherence to the treatment. Good compliance was defined as consuming more than 85% of the sachets, whereas any participant who missed more than 15% of the recommended doses were excluded from the final results.

### Drugs

The sachets containing probiotics supplemented with smectite (Symbiter Forte) or placebo had similar organoleptic characteristics, appearance, texture, weight, and smell. The only difference was on specified number code on them. The “Symbiter Forte” and the placebo were produced and delivered to the study center by “N.D. Prolisok” (Ukraine). The intervention contained a biomass of alive probiotic microorganism symbiosis, colony forming units - CFU/g: *Lactobacillus* – 1.0х10^9^, *Bifidobacterium* – 1.0х10^9^, *Lactococcus* – 1.0х10^8^, *Propionibacterium* – 1.0х10^8^ and *Acetobacter* – 1.0х10^5^; and smectite gel (250 mg). The microbial composition characterized with following richness: 17 strains of microorganisms physiological for mammalian intestines belonging to 11 species. Most strains were taken from All-union Collection of Industrial Microorganisms (ACIM) State Research Institute of Genetics and Breeding of Industrial Microorganisms (Union of Soviet Socialist Republics, Moscow): *Lactococcus lactic ssp. lactis* ACIM B-4305, ACIM B-5387; *Lactococcus lacti* ssp. diacetylactis ACIM B-4303; *Streptococcus salivarius ssp. thermophilus* ACIM B-4304, ACIM B-4741; *Lactobacillus acidophilus* ACIM B-5254, B-5863; *Lactobacillus delbrueckii ssp. bulgaricus* ACIM B-3963, B-5810; *Propionibacterium freudenreichii ssp. schermanii* ACIM B-4544; *Propionibacterium acidipropionici* ACIM B-5800; *Bifidobacterium bifidum* ACIM B-5799; *Bifidobacterium longum* ACIM B-4557, 4635; *Acetobacter aceti* ACIM B-5495. Also two strains were taken from depositary of D.K. Zabolotny Institute of Microbiology and Virology (IMV) of the National Academy of Sciences of Ukraine (Ukraine, Kyiv): *Lactobacillus helveticus* IMV B-7115; *Bifidobacterium bifidum* IMV B-7113).

We chose this probiotic based on our previous comparative experimental analysis for impact of different probiotic strains diabetes/obesity models. In this animal study, we assess beneficial effects of lyophilized mono-probiotic (*B. animalis VKL*, *B. animalis VKB*, *L.casei IMVB-7280*), the combination of this three strains and multiprobiotic “Symbiter” containing biomass of 17 alive probiotic strains. We have shown that supplementation of probiotic composition, with preference to alive strains, led to a significantly lower prevalence of obesity, improvement of IR, reduction of visceral adipose tissue weight and serum lipid levels as compared to single-strain probiotic (23, 24).

Every patient after randomization received 1 pack (10 g) of probiotic-smectite or placebo per day (QD) for 8-week period. All the participants were instructed concerning the use of the supplementation, i.e. they were told to cut the pack as shown, then dissolve the contents in 15 to 30 ml of boiled drinking water of ambient temperature, stir thoroughly and consume immediate after making ready.

### Outcomes assessment and measurement

After obtaining informed consent from the patients, they were requested to provide blood serum samples while fasting. The samples were then promptly frozen at t - 20°C. For each patient, relevant clinical and demographic data were collected.

The primary outcome was the assessment of β-cell function as change C-peptide and HOMA2-β (% B, homeostasis model assessment-estimated β-cell function) which was calculated using HOMA2 calculator. This model can be calculated using the software provided by the Oxford Centre for Diabetes, Endocrinology and Metabolism and available at http://www.dtu.ox.ac.uk/homacalculator/index.php. In addition to that insulin sensitivity (% S) and HOMA-2 index were its secondary outcome. C-peptide was measured using chemiluminescence immune analysis with the help of commercially available sets (Immulite, Siemens AG, Germany) with ng/ml scale.

The secondary outcomes of the RCT that were considered for investigating the efficiency of the probiotic-smectite therapy were glycemic control-related parameters, anthropometric variables and markers of a chronic systemic inflammatory response (TNF-α, ІL-1β, ІL-6, ІL-8, IFN-γ). All parameters were determined following a 12-h fasting period, by the hospital clinical laboratory.

The glucose level in the fasting state was determined by enzymatic Trinder method using an Exan device. HbA1c was measured using immunoturbidimetric analysis on Cobas 6000 (Roche Diagnostics, Basel, Switzerland) with a reference range of 4.8% to 5.9%. HbA1c level was standardized with a reference method in keeping with DCCT (Diabetes Control and Complications Trial) and NGSP (National Glycohemoglobin Standardization Program).

The level of pro-inflammatory cytokines was determined using enzyme-immunoassay with commercially available systems “Sigma” (TNF-α (RAB0476), ІL-1β (RAB0273), ІL-6 (RAB0306), ІL-8 (RAB0320), INF-γ (RAB0222)). Blood samples (5 ml) were taken in a fasting state. Serum was stored at t=−20°С. Cytokine levels under consideration were measured in each sample and expressed in pg/ml.

All the patients underwent anthropometry with the following data accumulated: body height (BH) accurate to 0.001 m; body weight (BW) accurate to 0.001 kg using medical scales. Body mass index (BMI) was calculated by Quetelet formula:


$$\text{BMI}=\text{BW}/{\text{BH}}^{2}$$

Waist circumference (WC) was measured using a flexible tape at the belly button level accurate to 0.001 m.

### Statistical analysis

Statistical analysis was done using a standard software SPSS version 20.0 (SPSS, Inc., Chicago, Illinois) and GraphPad Prism, version 6.0 (GraphPad Sofware, Inc., La Jolla, CA, USA). Quantitative changes were presented as the mean and standard deviation (М ± SD), qualitative changes were presented as %. To prove the normal distribution hypothesis, Kolmogorov-Smirnov one-sample test was used. To estimate the difference of the incoming quantitative data χ2 criterion was used. The changes in outcomes of the participants after the initiation of therapy and end of the trial were compared by paired sample t-tests (intra-group). Analysis of covariance (ANCOVA) was used to identify any differences between the two groups after intervention, adjusting for baseline measurements and confounders (BMI and sex) (inter-group).

## Results

**Figure 1:**
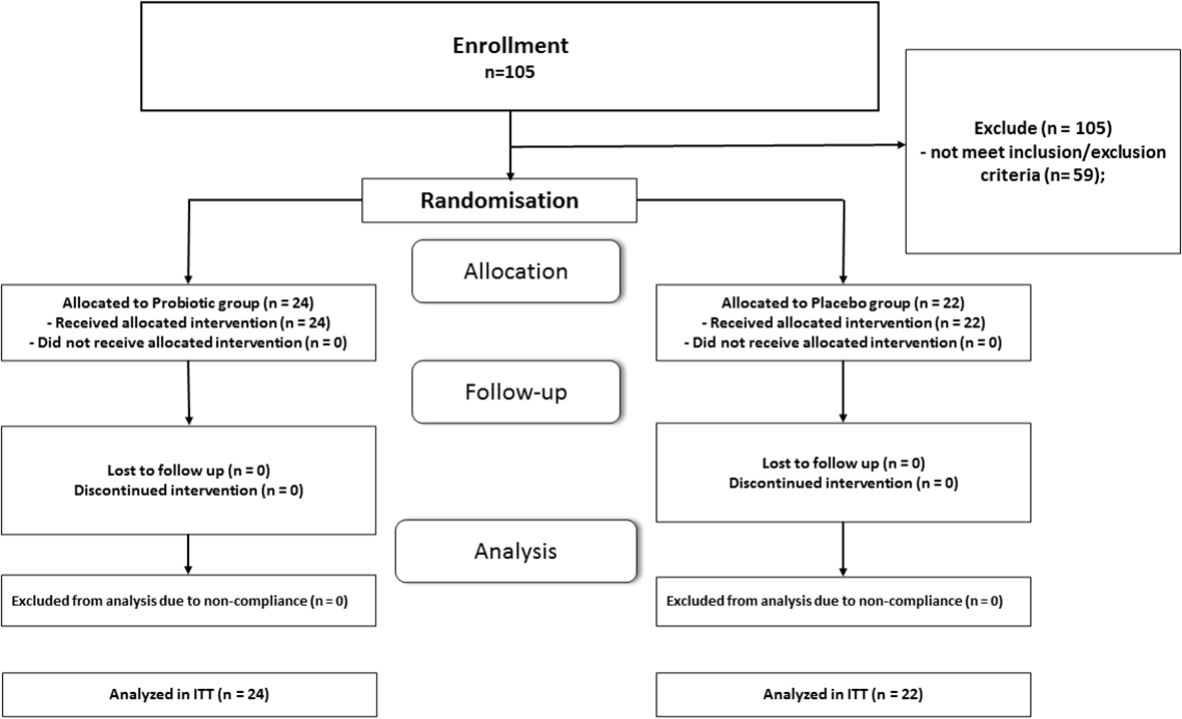
Consolidated Standards of Reporting Trials (CONSORT) flow chart-trial protocol.

Totally 105 patients with T2D completed primary RCT and their data were included in intention-to-treat analysis (19, 20). At initial stage we set as inclusion criteria %B as less than 50. From 105 patients only 28 meet the given criterion. After additional analysis of RCT database we found that 18 patients with T2D have %B in range 50-60%. Therefore, at the next meeting of the researchers, a decision was made to extend inclusion criterion % B to less than 60. After the specified modification of the protocol 46 patents meet inclusion/exclusion criteria for the secondary analysis, which greatly increased the statistical power of the study. Twenty-two patients received placebo and twenty-four were included in probiotic-smectite group. The CONSORT Flow Diagram is shown in **Figure 1**. All participants received standard care that included medical counseling, education in T2D, and lifestyle advice. All patients received more than 90% of the prescribed sachets in the double-blind treatment. The patients were satisfied with the organoleptic features; both additives were tolerated well. The main reported adverse events (AEs) were gastrointestinal complaints. In general, prevalence of AE was less than 20%, mild in their intensity and disappeared spontaneously (19, 20).

The baseline clinical parameters (age, gender and T2D duration) of the patients who completed the study are summarized in **Table 1**. All project participants were on standard treatment, namely oral hypoglycemic drugs, insulin, or combinations. Even though metformin can change the gut microbiota composition (25, 26), we did not exclude patients who took this drug in this study because metformin is currently recognized as a first-line therapy in T2D patients. Participants were randomized to the proportional shares of the patients with a stable metformin dosage 4 weeks before the study started. The two groups had no significant differences in the daily average amounts (**Table 1**).

**Table 1:** Baseline clinical parameters in examined patients (M ± SD or %).

Parameters	Placebo group (n=22)	Probiotic-Smectite group (n=24)	p
**Age, years**	54.87 ± 8.59	54.75 ± 8.87	0.971
**Duration of T2D, years**	13.53 ± 7.97	12.06 ± 6.18	0.569
**Metformin, % (n)**	59.1 (13)	62.5 (15)	0.813
**Metformin daily dosage, mg**	1495.0 ± 570.79	1636.36 ± 638.78	0.601
**Sulfonilureas, % (n)**	31.8 (7)	37.5 (9)	0.686
**Insulin daily dosage, IU**	31.66 ± 8.54	30.75 ± 7.92	0.839
**Insulinotherapy duration, years**	7.33 ± 4.84	7.75 ± 5.44	0.885
**Insulinotherapy, % (n)**	45.5 (10)	50.0 (12)	0.758

Analysis of primary and secondary outcomes of carbohydrate metabolism is presented in **Table 2**. At placebo group had no significant differences in carbohydrate metabolism parameters, except deterioration by 3% HbA1c (p=0.039), which a decrease in sensitivity (%S) and HOMA-2 can explain. Unfortunately, after 8 weeks of probiotic co-supplementation with absorbent smectite administration, we observed only a tendency for improving β-cell function (44.22 ± 12.80 *vs* 55.69 ± 25.75; р=0.094). Also, ANCOVA analysis didn’t show significant differences between the mean changes of the %B (-7.05 ± 21.4 *vs* -11.47 ± 26.04; p=0.642). The C-peptide concentration didn’t have statistically significant changes in both groups (**Table 2**).

**Table 2:** Analysis of primary and secondary outcomes with focus carbohydrate metabolism parameters (M ± SD).

Parameters	Placebo group (n=22)	p1	Probiotic-Smectite group (n=24)	p2	p3	p4
B-cell function, %B						
Baseline value Post-treatment value Mean changes	43.00 ± 10.70 50.05 ± 20.22 -7.05 ± 21.4	0.223	44.22 ± 12.80 55.69 ± 25.75 -11.47 ± 26.04	0.094	0.777	0.642
C-peptide, ng/ml						
Baseline value Post-treatment value Mean changes	3.11 ± 0.70 3.25 ± 0.91 -0.14 ± 0.51	0.372	3.33 ± 1.05 3.02 ± 1.10 0.31 ± 0.8	0.186	0.336	0.133
Glucose, mmol/l						
Baseline value Post-treatment value Mean changes	11.11 ± 1.57 10.70 ± 2.19 0.41 ± 2.70	0.567	11.44 ± 2.11 9.81 ± 2.29 1.63 ± 2.45	0.019	0.633	0.201
HbA1c, %						
Baseline value Post-treatment value Mean changes	8.2 ± 0.86 8.46 ± 0.99 -0.26 ± 0.45	0.039	8.56 ± 1.17 8.15 ± 1.14 0.41 ± 0.52	0.007	0.338	0.001
Sensitivity, %S						
Baseline value Post-treatment value Mean changes	35.53 ± 8.30 35.34 ± 8.59 0.19 ± 5.38	0.891	33.42 ± 10.67 41.00 ± 15.32 -7.58 ± 9.20	0.005	0.546	0.010
HOMA-2						
Baseline value Post-treatment value Mean changes	2.95 ± 0.64 3.00 ± 0.77 -0.05 ± 0.45	0.643	3.29 ± 1.03 2.74 ± 0.98 0.55 ± 0.65	0.003	0.285	0.006

p1-2 - difference in placebo and probiotic-smectite groups before and after intervention (intragroup analysis); p3 - differences between placebo and probiotic-smectite groups baseline characteristics; p4 - difference between groups throughout the study (ANCOVA intergroup analysis). Significance was stated at р<0,05.

However, the effectiveness of the therapy in the probiotic-smectite group was confirmed by other indicators. After 8 weeks of intervention fasting glycemia decreased by 14% (p=0.019), HbA1c – 5% (p=0.007), HOMA-2 – 17% (p=0.003) and increase of insulin sensitivity by 23% (p=0.005). Changes were significant also in inter-group analysis. The mean changes for both groups in ANCOVA analysis were as follow: HbA1c (-0.26 ± 0.45 *vs* 0.41 ± 0.52; p=0.001), %S (0.19 ± 5.38 *vs* -7.58 ± 9.20; р=0.01) and HOMA-2 (-0.05 ± 0.45 *vs* 0.55 ± 0.65; р=0.006) (**Table 2**).

Both groups’ anthropometric parameters changed insignificantly after the intervention (**Table 3**). The effect of probiotics and absorbent smectite therapy was also analyzed using its influence on the immune system. In the placebo group, all studied parameters did not change significantly. Analysis of the cytokine profile showed that statistical differences after treatment were in the concentration of both pro-inflammatory cytokines: IL-1β (22.83 ± 9.04 *vs* 19.03 ± 5.57; p=0.045) and TNF-α (31.25 ± 11.32 *vs* 26.23 ± 10.13; p=0.041). In the probiotic-smectite group, IL-6, IL-8, and γ-INF concentrations didn’t change significantly (**Table 4**). The between-group ANCOVA analysis didn’t find a change in all investigated cytokines.

**Table 3:** Analysis of anthropometric secondary outcomes (M ± SD).

Parameters	Placebo group (n=22)	p1	Probiotic-Smectite group (n=24)	p2	p3	p4
BMI, kg/m^2^						
Baseline value Post-treatment value Mean changes	33.61 ± 7.12 33.54 ± 6.88 0.07 ± 0.44	0.511	31.62 ± 4.96 31.56 ± 5.07 0.06 ± 0.32	0.431	0.371	0.933
Weight, kg						
Baseline value Post-treatment value Mean changes	97.17 ± 22.58 96.89 ± 21.67 0.28 ± 1.29	0.419	95.4 ± 17.32 95.05 ± 17.06 0.35 ± 1.06	0.213	0.808	0.870
WC, cm						
Baseline value Post-treatment value Mean changes	96.93 ± 6.00 96.93 ± 5.87 0.001 ± 1.10	0.999	95.81 ± 8.82 95.40 ± 8.59 0.59 ± 1.11	0.109	0.684	0.267

p1-2 - difference in placebo and probiotic-smectite groups before and after intervention (intragroup analysis); p3 - differences between placebo and probiotic-smectite groups baseline characteristics; p4 - difference between groups throughout the study (ANCOVA intergroup analysis). Significance was stated at р<0,05.

**Table 4:** Cytokine profile in patients with T2D (M ± SD).

Parameters	Placebo group (n=22)	p1	Probiotic-Smectite group (n=24)	p2	p3	p4
IL-1β, pg/ml						
Baseline value Post-treatment value Mean changes	20.38 ± 8.17 19.90 ± 6.93 0.48 ± 6.19	0.768	22.83 ± 9.04 19.03 ± 5.57 3.80 ± 6.94	0.045	0.437	0.170
TNF-α, pg/ml						
Baseline value Post-treatment value Mean changes	35.57 ± 9.33 35.18 ± 8.12 0.39 ± 7.80	0.848	31.25 ± 11.32 26.23 ± 10.13 5.01 ± 8.94	0.041	0.257	0.137
IL-8, pg/ml						
Baseline value Post-treatment value Mean changes	71.33 ± 23.25 69.62 ± 22.74 1.71 ± 10.36	0.534	78.06 ± 21.32 72.20 ± 20.54 5.86 ± 11.71	0.064	0.407	0.306
IL-6, pg/ml						
Baseline value Post-treatment value Mean changes	15.29 ± 7.52 15.19 ± 9.42 0.10 ± 5.89	0.949	21.30 ± 9.24 18.15 ± 7.12 3.15 ± 6.99	0.092	0.057	0.201
γ-INF, pg/ml						
Baseline value Post-treatment value Mean changes	160.54 ± 54.52 150.0 ± 66.09 10.54 ± 35.89	0.274	184.46 ± 55.40 177.40 ± 52.20 7.05 ± 28.75	0.342	0.236	0.767

p1-2 - difference in placebo and probiotic-smectite groups before and after intervention (intragroup analysis); p3 - differences between placebo and probiotic-smectite groups baseline characteristics; p4 - difference between groups throughout the study (ANCOVA intergroup analysis). Significance was stated at р<0,05.

## Discussion

In recent years, more scientific data have pointed out the close connection between intestinal microbial community, nutritional habits, lifestyle, and the appearance of various afflictions in the digestive tract, including irritable bowel syndrome (IBS) (27), metabolic diseases and cancer (28). Also, gut dysbiosis enhances the formation and accumulation of specific metabolites with toxic potential that induce the appearance of kidney-associated illnesses (29). Recent studies have shown that altering gut microbiota composition by probiotics, prebiotics and synbiotics can positively treat irritable bowel syndrome (IBS) (27).

Probiotics have been used safely in foods and dairy products for over a hundred years. Recently, there has been increasing interest in their use to prevent, mitigate or treat specific diseases (30). Changes in the gut microbiota composition and its derived metabolites are closely associated with insulin sensitivity (31), and energy homeostasis (32). Thus, the gut microbiota has been attracting much attention in metabolic diseases (33). Today, many clinical trials have investigated probiotics for metabolic diseases such as obesity and T2D (6, 33, 34). It was shown that changes in insulin secretion after lifestyle intervention (3 months of the nutritional program rich in fiber and exercise training) might be mediated via alterations in an insulinotropic polypeptide (GIP) secretion from intestinal K-cells (35).

This group of agents participates in several links of lipid (fat) metabolism at once, helping to accelerate the weight loss process and reduce the toxic load of harmful substances on the body. In this study, the probiotic was combined with absorbent smectite. Smectite (bentonite) is a natural loamy polymineral formed by microscopic particles capable of hydration and displays the most physiologically active properties. The smectite present on the surface of the intestinal tract provides cytomuco-protective therapeutic benefits by supplying energy and plastic materials to epithelial cells. This enhances the strength of the mucosal barrier and facilitates interaction between mineral particles and glycoproteins of the mucosa and the microbial biolayer (36). Smectite is unique in its ability to directly absorb viruses, toxins, radionuclides, heavy metals, and bacterial endotoxins without affecting normal microbiota cells or essential nutrients (19). Due to their absorbent activity and stabilization mucus layer properties, probiotics with smectite can impact the synergistic enhancement of a single effect, significantly increasing insulin sensitivity, improving anthropometric indicators and reducing inflammation (19).

The scientific data regarding smectite administration alone in terms of different metabolic disturbances are very scarce. Smectite has been effectively used in the treatment of several gastrointestinal diseases, including infectious diarrhea and food allergy (37). In experimental colitis models was found that administration of smectite associated with absorption of inflammatory proteins, reduction in systemic markers of inflammation (IL-2, IL-6, IL-8 and IL-12, TNFα, IFNγ) (38–40) and significant improvement in intestinal microbial profile (41). The Dening et al. proposed that spray dried smectite clay particles may be developed as novel anti-obesity treatments (42, 43). These particles had adsorptive capacities for dietary lipids and digestion products (42). When co-administered with a high-fat diet (HFD) to Sprague-Dawley rats, smectite clay particles can reduced the extent of weight gain relative to the negative control treatment group and performed similarly to orlistat *via* an alternate mechanism of action (43).

The systematic analysis of RCTs has supported the potential beneficial effects of metformin in improving β-cell function. This is especially true when it is combined with other anti-diabetic therapies like rosiglitazone, pioglitazone, and vildagliptin. One of the mechanisms behind this improvement is the reduction of pro-inflammatory markers like hs-CRP and TNF-α in patients with T2D (44). Our research aims to develop therapies that have antioxidant properties and reduce inflammation and oxidative stress markers to limit pancreatic β-cell failure in T2D patients and improve blood glucose control.

So, this study investigated the efficacy of probiotics and absorbent smectite for protecting against β-cells damage in T2D patients. Obesity can lead to the expansion of adipose tissue, which in turn produces several pro-inflammatory markers that hasten β-cell dysfunction. These effects are indicative of a compromised immune response, which exacerbates IR and raises blood glucose levels. Concurrently, oxidative stress occurs alongside inflammation, causing disruptions in various biochemical processes that can lead to the death of pancreatic β-cells (44). After 8 weeks of probiotic co-supplementation with absorbent smectite administration, only a tendency for improving β-cell function as a change in %B was found. However, in this group, there was decreased fasting glycemia HbA1c parallel with improvement of insulin sensitivity. Bacteria in the colon use undigested dietary substrates from the small intestines for survival. Carbohydrates fermentation by bacteria produces beneficial metabolites. However, in the case of carbohydrates limitation, bacteria can use alternative energy sources and produce different metabolites, which have a harmful effect on human health. Dietary carbohydrates’ main bacterial fermentation products are short-chain fatty acids (SCFAs) and gases (45). Recent studies found that a ligand of toll-like receptor (TLR) 4, activated by metabolites gut microbiota, plays an essential role in the development of IR and obesity as well as in oxidative stress, inflammation, cell proliferation, and apoptosis (46–48). In our study, we also analyzed the effect of the applied therapy on the immune system. Still, the concentration of both pro-inflammatory cytokines were decreased: IL-1β by 17% (p=0.045) and TNF-α by 16% (p=0.041).

In this placebo-controlled RCT, patients were on standard therapy with insulin or oral anti-diabetic drugs, primarily metformin. As mentioned above, patients with metformin were randomly divided into two groups. It has shown that metformin can inhibit microbial bile acids’ metabolism by altering gut microbiome symbiosis and blocking gut bile acids’ signaling, thereby partially exerting their metabolic benefits (49).

Western diet, which contains a lot of sugars and fat, including trans-fatty acids and cholesterol, leads to dysbiosis. Such a HFD connected with reduced expression of tight junction genes results in increased intestinal permeability. Raised intestinal mucosa permeability with loss of integrity facilitates enteric bacterial pathogens that contain lipopolysaccharides (LPS). Moreover, in mice, LPS increases intestinal permeability both *in vitro* and *in vivo*, suggesting an association between increased intestinal LPS level and the expression of gut tight junction (50, 51). Increased intestinal permeability may contribute to a more significant effect on the immune system of pathogenic antigens and diet, causing mild chronic systemic inflammation and immune-mediated destruction of pancreatic β-cells, which ultimately causes T2D (52–54). That’s why sorbents are essential in the fight against excess weight and T2D.

The main limitation of this study was unidentified composition of the intestinal microbiota in the patients at the baseline and post-treatment state with view of defining the personalized impact upon the changes of metabolic parameters. Furthermore, the terms of treatment for 8 weeks may not fully reproduce the changes of HbA1c as it the glycosylation status of RBCs from around 120 days prior to the test date. The latter should be taken into consideration in future studies.

## Conclusion

It was found that incorporating a live multistrain probiotic and absorbent smectite supplement positively impacted β-cell function and parallel with reduction of glycemic related parameters in individuals with T2D. This discovery indicates that modifying the gut microbiota could potentially be a successful diabetes treatment and should be explored in more extensive studies.

## Data availability statement

The raw data supporting the conclusions of this article will be made available by the authors, without undue reservation.

## Ethics statement

The studies involving humans were approved by Local Ethics Committee at Kyiv City Clinical Endocrinology Centre. The studies were conducted in accordance with the local legislation and institutional requirements. The participants provided their written informed consent to participate in this study.

## Author contributions

MS: Conceptualization, Data curation, Investigation, Methodology, Project administration, Resources, Supervision, Writing – original draft, Writing – review & editing. DK: Data curation, Investigation, Writing – review & editing. GZ: Formal Analysis, Methodology, Writing – review & editing. DO: Formal Analysis, Methodology, Writing – review & editing. TF: Conceptualization, Writing – original draft, Writing – review & editing. NK: Conceptualization, Investigation, Project administration, Resources, Visualization, Writing – original draft, Writing – review & editing.

## References

[1] Ong KL Stafford LK McLaughlin SA Boyko EJ Vollset SE Smith AE. Global, regional, and national burden of diabetes from 1990 to 2021, with projections of prevalence to 2050: a systematic analysis for the Global Burden of Disease Study 2021. Lancet (London England) (2023) 402:203–34. doi: 10.1016/S0140-6736(23)01301-6 PMC10364581 37356446

[2] Yousri NA Suhre K Yassin E Al-Shakaki A Robay A Elshafei M. Metabolic and metabo-clinical signatures of type 2 diabetes, obesity, retinopathy, and dyslipidemia. Diabetes (2022) 71:184–205. doi: 10.2337/db21-0490 34732537 PMC8914294

[3] Marushchak M Kozak K Krynytska I. Comorbid overweight/obesity and chronic pancreatitis exacerbate the dyslipidemia progression in type 2 diabetic patients. Endocr Regul (2022) 56:168–77. doi: 10.2478/enr-2022-0018 35843717

[4] Muzurović E Kraljević I Solak M Dragnić S Mikhailidis DP. Homocysteine and diabetes: Role in macrovascular and microvascular complications. J Diabetes Complicat (2021) 35:107834. doi: 10.1016/j.jdiacomp.2020.107834 33419630

[5] DeMarsilis A Reddy N Boutari C Filippaios A Sternthal E Katsiki N. Pharmacotherapy of type 2 diabetes: An update and future directions. Metabolism (2022) 137:155332. doi: 10.1016/j.metabol.2022.155332 36240884

[6] Kobyliak N Falalyeyeva T Kyriachenko Y Tseyslyer Y Kovalchuk O Hadiliia O. Akkermansia muciniphila as a novel powerful bacterial player in the treatment of metabolic disorders. Minerva Endocrinol (2022) 47:242–52. doi: 10.23736/S2724-6507.22.03752-6 35103461

[7] Falalyeyeva T Mamula Y Scarpellini E Leshchenko I Humeniuk A Pankiv I. Probiotics and obesity associated disease: an extended view beyond traditional strains. Minerva Gastroenterol (2021) 67:348–56. doi: 10.23736/S2724-5985.21.02909-0 35040301

[8] Hijová E. Synbiotic supplements in the prevention of obesity and obesity-related diseases. Metabolites (2022) 12:313. doi: 10.3390/metabo12040313 35448499 PMC9031884

[9] Mykhalchyshyn G Kobyliak N Bodnar P. Diagnostic accuracy of acyl-ghrelin and it association with non-alcoholic fatty liver disease in type 2 diabetic patients. J Diabetes Metab Disord (2015) 14:44. doi: 10.1186/s40200-015-0170-1 25995986 PMC4438435

[10] Scheithauer TPM Rampanelli E Nieuwdorp M Vallance BA Verchere CB van Raalte DH. Gut microbiota as a trigger for metabolic inflammation in obesity and type 2 diabetes. Front Immunol (2020) 11:571731. doi: 10.3389/fimmu.2020.571731 33178196 PMC7596417

[11] Korotkyi O Vovk A Galenova T Vovk T Dvorschenko K Luzza F. Effect of probiotic on serum cytokines and matrix metalloproteinases profiles during monoiodoacetate-induced osteoarthritis in rats. Minerva Biotecnol (2019) 31:68–73. doi: 10.23736/S1120-4826.19.02548-5

[12] Chen L Deng H Cui H Fang J Zuo Z Deng J. Inflammatory responses and inflammation-associated diseases in organs. Oncotarget (2018) 9:7204–18. doi: 10.18632/oncotarget.23208 PMC5805548 29467962

[13] Kobyliak N Abenavoli L Falalyeyeva T Beregova T. Efficacy of probiotics and smectite in rats with non-alcoholic fatty liver disease. Ann Hepatol (2018) 17:153–61. doi: 10.5604/01.3001.0010.7547 29311399

[14] Eguchi K Nagai R. Islet inflammation in type 2 diabetes and physiology. J Clin Invest (2017) 127:14–23. doi: 10.1172/JCI88877 28045399 PMC5199688

[15] Ying W Fu W Lee YS Olefsky JM. The role of macrophages in obesity-associated islet inflammation and β-cell abnormalities. Nat Rev Endocrinol (2020) 16:81–90. doi: 10.1038/s41574-019-0286-3 31836875 PMC8315273

[16] Eguchi N Vaziri ND Dafoe DC Ichii H. The role of oxidative stress in pancreatic β Cell dysfunction in diabetes. Int J Mol Sci (2021) 22:1–18. doi: 10.3390/IJMS22041509 PMC7913369 33546200

[17] Hasain Z Mokhtar NM Kamaruddin NA Mohamed Ismail NA Razalli NH Gnanou JV. Gut microbiota and gestational diabetes mellitus: A review of host-gut microbiota interactions and their therapeutic potential. Front Cell Infect Microbiol (2020) 10:188. doi: 10.3389/fcimb.2020.00188 32500037 PMC7243459

[18] Bahman Y Maryam M Aisa B Falalyeyeva T Kobyliak N Majid E. Immunomodulatory role of Faecalibacterium prausnitzii in obesity and metabolic disorders. Minerva Biotechnol Biomol Res (2021) 33:76–85. doi: 10.23736/S2724-542X.21.02759-2

[19] Kobyliak N Abenavoli L Falalyeyeva T Kovalchuk O Kyriienko D Komisarenko I. Metabolic benefits of probiotic combination with absorbent smectite in type 2 diabetes patients a randomised controlled trial. Rev Recent Clin Trials (2021) 16:109–19. doi: 10.2174/1574887115666200709141131 32646362

[20] Kobyliak N Abenavoli L Mykhalchyshyn G Falalyeyeva T Tsyryuk O Kononenko L. Probiotics and smectite absorbent gel formulation reduce liver stiffness, transaminase and cytokine levels in NAFLD associated with type 2 diabetes: A randomized clinical study. Clin Diabetol (2019) 8:205–14. doi: 10.5603/DK.2019.0016

[21] American Diabetes Association. 2. Classification and diagnosis of diabetes: Standards of medical care in diabetesd2019. Diabetes Care (2019) 42:S13–28. doi: 10.2337/dc19-S002 30559228

[22] Kobyliak N Khomenko M Falalyeyeva T Fedchenko A Savchuk O Tseyslyer Y. Probiotics for pancreatic β-cell function: from possible mechanism of action to assessment of effectiveness. Crit Rev Microbiol (2023). doi: 10.1080/1040841X.2023.2257776 37705353

[23] Kobyliak N Falalyeyeva T Virchenko O Mykhalchyshyn G Bodnar P Spivak M. Comparative experimental investigation on the efficacy of mono- and multiprobiotic strains in non-alcoholic fatty liver disease prevention. BMC Gastroenterol (2016) 16:34. doi: 10.1186/s12876-016-0451-2 26976285 PMC4791938

[24] Kobyliak N Falalyeyeva T Beregova T Spivak M. Probiotics for experimental obesity prevention: Focus on strain dependence and viability of composition. Endokrynol Polska (2017) 68:659–67. doi: 10.5603/EP.a2017.0055 29022648

[25] Kyriachenko Y Falalyeyeva T Korotkyi O Molochek N Kobyliak N. Crosstalk between gut microbiota and antidiabetic drug action. World J Diabetes (2019) 10:154–68. doi: 10.4239/wjd.v10.i3.154 PMC6422856 30891151

[26] Montandon SA Jornayvaz FR. Effects of antidiabetic drugs on gut microbiota composition. Genes (2017) 8:250. doi: 10.3390/genes8100250 28973971 PMC5664100

[27] Simon E Călinoiu LF Mitrea L Vodnar DC. Probiotics, prebiotics, and synbiotics: implications and beneficial effects against irritable bowel syndrome. Nutrients (2021) 13:2112. doi: 10.3390/nu13062112 34203002 PMC8233736

[28] Fernández-Millán E Guillén C. Multi-organ crosstalk with endocrine pancreas: A focus on how gut microbiota shapes pancreatic beta-cells. Biomolecules (2022) 12:104. doi: 10.3390/biom12010104 35053251 PMC8773909

[29] Mitrea L Medeleanu M Pop C-R Rotar A-M Vodnar D-C. Biotics (Pre-, pro-, post-) and uremic toxicity: implications, mechanisms, and possible therapies. Toxins (2023) 15:548. doi: 10.3390/toxins15090548 37755974 PMC10535688

[30] Doron S Snydman DR. Risk and safety of probiotics. Clin Infect Dis (2015) 60:S129–34. doi: 10.1093/cid/civ085 PMC4490230 25922398

[31] De Vadder F Kovatcheva-Datchary P Goncalves D Vinera J Zitoun C Duchampt A. Microbiota-generated metabolites promote metabolic benefits via gut-brain neural circuits. Cell (2014) 156:84–96. doi: 10.1016/j.cell.2013.12.016 24412651

[32] Kimura I Inoue D Maeda T Hara T Ichimura A Miyauchi S. Short-chain fatty acids and ketones directly regulate sympathetic nervous system via G protein-coupled receptor 41 (GPR41). Proc Natl Acad Sci (2011) 108:8030–5. doi: 10.1073/pnas.1016088108 PMC3093469 21518883

[33] Falalyeyeva T Chornenka N Cherkasova L Tsyryuk O Molchek N Kovalchuk O. Gut microbiota interactions with obesity. In: Reference Module in Food Science. Amsterdam, Netherlands: Elsevier (2022). doi: 10.1016/B978-0-12-819265-8.00030-9

[34] Kanazawa A Aida M Yoshida Y Kaga H Katahira T Suzuki L. Effects of synbiotic supplementation on chronic inflammation and the gut microbiota in obese patients with type 2 diabetes mellitus: A randomized controlled study. Nutrients (2021) 13:558. doi: 10.3390/nu13020558 33567701 PMC7914668

[35] Solomon TPJ Haus JM Kelly KR Rocco M Kashyap SR Kirwan JP. Improved pancreatic β-cell function in type 2 diabetic patients after lifestyle-induced weight loss is related to glucose-dependent insulinotropic polypeptide. Diabetes Care (2010) 33:1561–6. doi: 10.2337/dc09-2021 PMC2890359 20200305

[36] Weese JS Cote NM Degannes RVG. Evaluation of in *vitro* properties of di-tri-octahedral smectite on clostridial toxins and growth. Equine Vet J (2010) 35:638–41. doi: 10.2746/042516403775696384 14649353

[37] Müller J Dobler D Schmidts T Rusch V. Smectite for medical use and their toxin binding capacity. J Food Nutr Popul Health (2019) 3:1–5. doi: 10.36648/2577-0586.3.1.16

[38] Zychowski KE Elmore SE Rychlik KA Ly HJ Pierezan F Isaiah A. Mitigation of colitis with NovaSil clay therapy. Digest Dis Sci (2015) 60:382–92. doi: 10.1007/S10620-014-3360-7 25240298

[39] González R Sánchez De Medina F Martínez-Augustin O Nieto A Gálvez J Risco S. Anti-inflammatory effect of diosmectite in hapten-induced colitis in the rat. Br J Pharmacol (2004) 141:951–60. doi: 10.1038/sj.bjp.0705710 PMC1574279 14993105

[40] Mahraoui L Heyman M Plique O Droy-Lefaix MT Desjeux JF. Apical effect of diosmectite on damage to the intestinal barrier induced by basal tumour necrosis factor-α. Gut (1997) 40:339–43. doi: 10.1136/gut.40.3.339 PMC1027083 9135522

[41] Breitrück A Weigel M Hofrichter J Sempert K Kerkhoff C Mohebali N. Smectite as a preventive oral treatment to reduce clinical symptoms of DSS induced colitis in Balb/c mice. Int J Mol Sci (2021) 22:8699. doi: 10.3390/IJMS22168699 34445403 PMC8395406

[42] Dening TJ Joyce P Kovalainen M Gustafsson H Prestidge CA. Spray dried smectite clay particles as a novel treatment against obesity. Pharm Res (2018) 36:21. doi: 10.1007/S11095-018-2552-9 30519891

[43] Joyce P Dening TJ Meola TR Wignall A Ulmefors H Kovalainen M. Contrasting anti-obesity effects of smectite clays and mesoporous silica in sprague-dawley rats. ACS Appl Bio Mater (2020) 3:7779–88. doi: 10.1021/ACSABM.0C00969 35019518

[44] Dludla PV Mabhida SE Ziqubu K Nkambule BB Mazibuko-Mbeje SE Hanser S. Pancreatic β-cell dysfunction in type 2 diabetes: Implications of inflammation and oxidative stress. World J Diabetes (2023) 14:130–46. doi: 10.4239/wjd.v14.i3.130 PMC10075035 37035220

[45] Million M Angelakis E Maraninchi M Henry M Giorgi R Valero R. Correlation between body mass index and gut concentrations of Lactobacillus reuteri, Bifidobacterium animalis, Methanobrevibacter smithii and Escherichia coli. Int J Obes (2013) 37:1460–6. doi: 10.1038/ijo.2013.20 PMC3826031 23459324

[46] Cani PD. Human gut microbiome: Hopes, threats and promises. Gut (2018) 67:1716–25. doi: 10.1136/gutjnl-2018-316723 PMC6109275 29934437

[47] Benomar Y Taouis M. Molecular mechanisms underlying obesity-induced hypothalamic inflammation and insulin resistance: pivotal role of resistin/TLR4 pathways. Front Endocrinol (2019) 10:140. doi: 10.3389/fendo.2019.00140 PMC6418006 30906281

[48] Hasan A Akhter N Al-Roub A Thomas R Kochumon S Wilson A. TNF-α in combination with palmitate enhances IL-8 production via the MyD88- independent TLR4 signaling pathway: potential relevance to metabolic inflammation. Int J Mol Sci (2019) 20:4112. doi: 10.3390/ijms20174112 31443599 PMC6747275

[49] Sun L Xie C Wang G Wu Y Wu Q Wang X. Gut microbiota and intestinal FXR mediate the clinical benefits of metformin. Nat Med (2018) 24:1919–29. doi: 10.1038/s41591-018-0222-4 PMC6479226 30397356

[50] Rohr MW Narasimhulu CA Rudeski-Rohr TA Parthasarathy S. Negative effects of a high-fat diet on intestinal permeability: A review. Adv Nutr (2020) 11:77–91. doi: 10.1093/advances/nmz061 31268137 PMC7442371

[51] Binienda A Twardowska A Makaro A Salaga M. Dietary carbohydrates and lipids in the pathogenesis of leaky gut syndrome: An overview. Int J Mol Sci (2020) 21:1–17. doi: 10.3390/ijms21218368 PMC7664638 33171587

[52] Knip M Siljander H. The role of the intestinal microbiota in type 1 diabetes mellitus. Nat Rev Endocrinol (2016) 12:154–67. doi: 10.1038/nrendo.2015.218 26729037

[53] Mejía-León ME Calderón de la Barca AM. Diet, microbiota and immune system in type 1 diabetes development and evolution. Nutrients (2015) 7:9171–84. doi: 10.3390/nu7115461 PMC4663589 26561831

[54] Sabatino A Regolisti G Cosola C Gesualdo L Fiaccadori E. Intestinal microbiota in type 2 diabetes and chronic kidney disease. Curr Diabetes Rep (2017) 17:16. doi: 10.1007/s11892-017-0841-z 28271466

